# Metabolic Heterogeneity of Obesity and Outcome-Specific Effects of GLP-1 Receptor Agonists in Cardiovascular-Kidney-Metabolic Syndrome

**DOI:** 10.1016/j.jacadv.2026.102808

**Published:** 2026-05-14

**Authors:** Laurent Fauchier, Olivier Morel

**Affiliations:** a Centre Hospitalier Universitaire Trousseau et Faculté de Médecine, Université François Rabelais, Tours, France; b University of Strasbourg, Strasbourg, France

We read with interest the State-of-the-Art Review by Kumar et al.^1^ on glucagon-like peptide-1 receptor agonists (GLP-1RAs) in cardiovascular-kidney-metabolic syndrome.

Although the review convincingly highlights the pleiotropic benefits of GLP-1RAsacross organ systems, greater emphasis on the metabolic heterogeneity of obesity could further refine the interpretation of cardiovascular risk and treatment effects.

In this context, the distinction between metabolically healthy obesity (MHO) and metabolically unhealthy obesity (MUHO) has been proposed, based on the absence or presence of obesity-related metabolic abnormalities such as diabetes, hypertension, or dyslipidaemia.^2^, ^3^, ^4^, ^5^ Importantly, cardiovascular risk associated with obesity differs markedly according to both metabolic status and clinical outcome.^2^ For atherosclerotic events, multiple large cohort studies have shown that individuals with MHO have little or no excess risk of myocardial infarction or ischemic stroke compared with metabolically healthy normal-weight individuals, whereas MUHO is consistently associated with a substantially increased risk. This pattern supports the concept that ischemic risk is largely driven by metabolic dysfunction rather than adiposity itself. In contrast, heart failure and atrial fibrillation display a different risk profile. Even in the absence of overt metabolic abnormalities, MHO is associated with a persistently higher risk of incident heart failure and atrial fibrillation, albeit lower than in MUHO, suggesting weight-related hemodynamic load, cardiac remodeling, and adipose tissue distribution as dominant mechanisms.^2^ Accordingly, the cardiovascular benefit of GLP-1RAs on ischemic outcomes appears most pronounced in MUHO, whereas observational data show more modest or nonsignificant associations in MHO, particularly for atrial fibrillation.^4^

These distinctions have direct therapeutic implications. In MUHO, GLP-1RAs may reduce cardiovascular events predominantly through improvement of metabolic abnormalities that drive atherosclerosis and adverse outcomes. In MHO, where baseline ischemic risk is lower but risks of heart failure and atrial fibrillation remain elevated, the role of GLP-1RAs may be more preventive, aimed at delaying progression to metabolic unhealthiness rather than producing immediate reductions in myocardial infarction or stroke.^3^^,^^4^ Explicitly integrating MHO and MUHO phenotypes—and recognizing outcome-specific risk patterns—within the cardiovascular-kidney-metabolic framework could therefore help personalize GLP-1RA use, align expectations regarding cardiovascular benefit, and strengthen the clinical applicability of this otherwise comprehensive review.
